# Supplementary material for: Identification of photoperiod-induced specific miRNAs in the adrenal glands of Sunite sheep (Ovis aries)
Source: Front Vet Sci. 2022 Jul 22;9:888207. doi: 10.3389/fvets.2022.888207 (PMC9354845; doi:10.3389/fvets.2022.888207)
Supplement: Supplementary file 1 [file Data_Sheet_1.ZIP › Supplementary file/miRDeep2 file.zip!/miRDeep2 file/Know_Structure/SPLP42A2/oar-mir-665.pdf]

miRBase precursor : oar-mir-665
Total read count : 77
oar-miR-665-5p read count : 3
oar-miR-665-3p read count : 69
remaining reads : 5

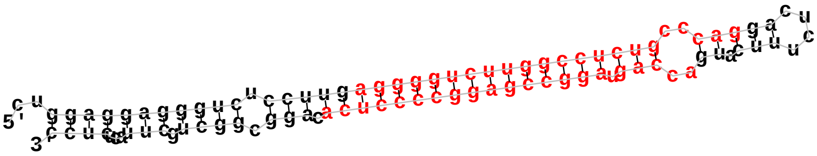

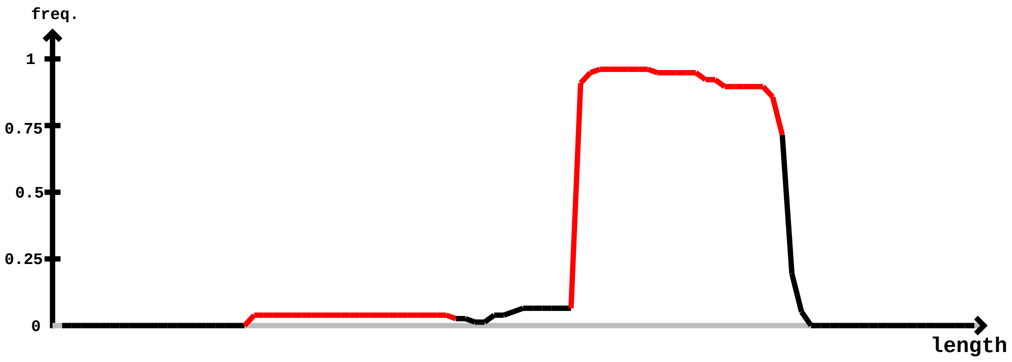


```
                                                                oar-miR-665-3p
                        oar-miR-665-5p
5'- cuggaggagggucuccuugaggggucuuggccucugcccaggacucuuucaugaccaguaggccgaggccccucacaggcggcugcuuacucucc   -3'   exp
    ..(((((((((((.((((((((((((((((((((((..(((((....))).))..))).)))))))))))))))).))).)))).))).))))..     reads      mm        sample
    ...................aggggucuuggccucugccc........................................................     1          0         seq
    ...................aggggucuuggccucugccca.......................................................     1          0         seq
    ...................aggggucuuggccucugcccagg.....................................................     1          0         seq
    .......................................aggacucuuucaugaccaguag..................................     1          0         seq
    ............................................ucuuucaugaccaguaggccga.............................     2          0         seq
    ..............................................uuucaugaccaguaggccgagg...........................     1          0         seq
    ...............................................uucaugaccaguaggccgagg...........................     1          0         seq
    .....................................................accaguaggccgaggccccu......................     3          0         seq
    .....................................................accaguaggccgaggccccuc.....................     10         0         seq
    .....................................................accaguaggccgaggccccuca....................     39         0         seq
    .....................................................accaguaggccgaggccccucac...................     9          0         seq
    .....................................................accaguaggccgaggccccucaca..................     4          0         seq
    ......................................................ccaguaggccgaggccccuc.....................     1          0         seq
    ......................................................ccaguaggccgaggccccuca....................     1          0         seq
    ......................................................ccaguaggccgaggccccucac...................     1          0         seq
    .......................................................caguaggccgaggccccucac...................     1          0         seq
```
